# Astragalus polysaccharides suppresses high glucose-induced metabolic memory in retinal pigment epithelial cells through inhibiting mitochondrial dysfunction-induced apoptosis by regulating miR-195

**DOI:** 10.1186/s10020-019-0088-z

**Published:** 2019-05-22

**Authors:** Ping Liu, Qing-Hua Peng, Ping Tong, Wen-Jie Li

**Affiliations:** 1 Department of Ophthalmology, The Second Xiangya Hospital of Central South University, Changsha, 410011 People’s Republic of China; 2 Hunan Provincial Key Laboratory of Ophthalmology and Otorhinolaryngology of Chinese Medicine, Changsha, 410007 People’s Republic of China; 3 Department of Ophthalmology, The Third Xiangya Hospital of Central South University, No.138, Tongzipo Road, Yuelu District, Changsha, 410013 Hunan Province People’s Republic of China

**Keywords:** Metabolic memory, Diabetic retinopathy, Mitochondrial damage, Apoptosis, Astragalus polysaccharides, miR-195

## Abstract

**Background:**

Metabolic memory contributes to the development of diabetic retinopathy (DR), which is the complication of diabetes. But it’s still unknown how to prevent the metabolic memory to treat the DR. In our study, we want to examine the function of Astragalus polysaccharides (APS) in the metabolic memory of retinal pigment epithelium (RPE) pretreated with high glucose (HG).

**Methods:**

ARPE-19 and PRPE cells were exposed to HG followed by normal glucose (NG) treatment with or without APS. QPCR was used to examine the levels of miR-195 and Bcl-2. MDA and SOD detection assays were used to examine the oxidative stress level. Western blotting and immunostaining were applied to detect the protein level of mitochondrial damage and apoptotic signaling pathway. Flow cytometry and TUNEL staining were used to analyze cell apoptosis. Luciferase assay was used to examine the direct target of miR-195.

**Results:**

APS treatment significantly decreased the expression of miR-195, while increased the expression of Bcl-2 with optimized dosages which were induced by HG treatment, even after replacing the HG with NG. And we found Bcl-2 was the direct target of miR-195. APS alleviated the oxidative stress, mitochondrial damage and cell apoptosis induced by HG and HG + NG treatments in RPE cells via regulating miR-195. Furthermore, we found overexpression of miR-195 abolished the alleviated effects of APS on the HG-treated RPE cells.

**Conclusions:**

APS suppressed high glucose-induced metabolic memory in retinal pigment epithelial cells through inhibiting mitochondrial dysfunction-induced apoptosis by regulating miR-195.

## Background

Diabetic retinopathy (DR), the leading cause of blindness in adults around the world, is one of the major vascular complications of diabetes that caused by long-term detrimental effects of hyperglycaemia (Brownlee 2001; Klein et al. 1994; Kowluru 2013). DR is a slow progressing disease which includes the gradual alterations of retinal microvasculature (Frank 2004). Hyperglycemia is the major factor for the development of DR (Klein 1995). Studies have found that DR persists even after the normalization of the blood glucose after hyperglycemia (Kowluru 2017). The Diabetes Control and Complications Trial (DCCT) and Epidemiology of Diabetes Interventions and Complications (EDIC) studies show hyperglycemia induces long-term deleterious consequences on diabetic complications, even after the strict glycemic control, which is defined as “metabolic memory” (Diabetes et al. 1993; Diabetes C, Complications Trial/epidemiology of Diabetes I, Complications Research G, et al. 2000). This indicates the early control of glycemic is important for DR (Writing Team for the Diabetes C, Complications Trial/Epidemiology of Diabetes I, Complications Research G 2002; Reddy et al. 2015). It has been proved that metabolic memory is related to the progression of DR (Zhang et al. 2012). Studies on dog models of DR showed the tight glycemic control after poor glycemic control didn’t arrest the progression of DR (Engerman and Kern 1995). Rat models of DR demonstrated early intervention by islet transplantation more effectively arrested the progression of DR than the late intervention (Hammes et al. 1993). Furthermore, in vitro studies also demonstrated that exposure to high glucose persistently altered the parameters of oxidative stress, inflammatory factors, DNA damages and mitochondria dysfunction (Kowluru 2013; Tewari et al. 2012; Zhong and Kowluru 2010; Zhong and Kowluru 2011; Zhang et al. 2015). All of these indicated metabolic memory played important roles in the development of DR. It’s important to study the other detailed mechanisms and seek effective drugs for metabolic memory to treat DR and other diabetic complications.

Astragalus polysaccharides (APS) is the one of the main bioactive components of *Astragalus membranaceus* (Huang Qi in China), which is Chinese traditional medicinal formulas for treating diabetes (Sang et al. 2010; Liu et al. 2013). Studies have shown that APS can effectively alleviate diabetes and diabetic complications via improving whole-body glucose homeostasis and increasing insulin sensitivity in skeletal muscle (Liu et al. 2013; Mao et al. 2007; Liu et al. 2010). However, the function of APS in metabolic memory is still unknown. Previous reports found APS ameliorated the mitochondrial dysfunction through Sirtunin1 pathway in chronic fatigue (Huang et al. 2016). Furthermore, studies showed mitochondrial damage also occurred in metabolic memory rat model (Kowluru et al. 2004a; Kowluru 2003). So we hypothesized APS could control the metabolic memory via regulating mitochondrial dysfunction.

The retinal pigment epithelium (RPE), a single layer of epithelial cells lying between the choroid and neurosensory retina, plays crucial roles in photoreceptor function, including oxidative stress response, photoreceptor renewal, phagocytosis of photoreceptor and preservation of photo transduction (Sparrow et al. 2010; Klettner 2012; Farnoodian et al. 2015). The RPE cells also selectively transport of metabolites, ions, nutrients and water between retina and choriocapillaris (Strauss 2005). The dysfunction of RPE is related to retinal degeneration and irreversible vision loss, and is the first event in DR (Yang et al. 2006; Farnoodian et al. 2016; Simao et al. 2017; Xie et al. 2014). In rodent diabetic models, hyperglycemia induced the dysfunction, even death of RPE cells which led to the development of DR (Xia and Rizzolo 2017). Furthermore, studies showed high glucose induced mitochondrial dysfunction and death in RPE (Li et al. 2012; Chen et al. 2016). These data suggested RPE cells might also have the metabolic memory effect. Previous study suggested RPE isolated from age-related macular degeneration (AMD) patients possessed the metabolic memory (Ferrington et al. 2017). All of these suggested illustration of the connection between the metabolic memory of RPE and DR might provide a novel therapeutic target for treatment of DR. Since APS can regulate mitochondrial damage, we want to examine the function of APS in the mitochondrial injury-induced apoptosis in RPE cells to explore the phenomenon of metabolic memory in DR.

MicroRNAs (miRNAs) are ~ 22 bp small non-coding RNA which can repress the expression of target genes via directly targeting to the 3′ untranslated region (UTR) of genes at the posttranscriptional level (Lim et al. 2003; Shukla et al. 2011). MiRNAs are involved in many diseases, like neurodegenerative diseases, cancers, cardiovascular diseases and diabetes et al. (Hata 2013; Zhang et al. 2009; Farazi et al. 2011; Saraiva et al. 2017; Ortega et al. 2014). MiR-195 is a miRNA which is highly expressed in diabetic tissues, including kidney, liver and heart (Zheng et al. 2015; Chen et al. 2011; Herrera et al. 2010). Studies also showed miR-195 was also highly expressed in retinal endothelial cells of diabetic rats and played important roles in DR (Mortuza et al. 2014; Zhang et al. 2017). However, the molecular mechanisms for the regulation of miR-195 in diabetes and DR are still elusive. Furthermore, miR-195 can directly repress the expression of Bcl-2 which is directly related to the cell apoptosis induced by the mitochondrial dysfunction in diabetes and breast cancer (Chen et al. 2011; Singh and Saini 2012; Alarifi et al. 2017). Thus, we wonder if control of metabolic memory by APS is through regulating the expression of miR-195.

In the current study, we established the metabolic memory model in the cultured RPE cells, and found APS treatment significantly repressed the mitochondrial damage-induced RPE cell apoptosis via regulating the expressions of miR-195 and Bcl-2. So we found APS was benefit for repressing the metabolic memory in RPE, which provided to explore the mechanisms and lay the theoretical foundation of searching effective therapeutic drugs in metabolic memory. Furthermore, it would provide a new therapeutic target for the treatment of DR.

## Methods

### Isolation and culture of rat primary RPE cells

Primary RPE cells (PRPE) were isolated from rat by removing retinas and optic nerve. Then, the remaining cup were cut to collect the RPE layer under a dissecting microscope. The RPE sheets were digested in 5 ml of 1 mg/ml Collagenase type I (Worthington) in 37 °C for 20 min. The digested tissues were washed with DMEM containing 10% FBS followed by resuspending in RPE cell growth medium (DMEM containing 10% FBS, 2 mM L-glutamin, 100 μg/ml streptomycin, 100 U/ml penicillin and murine recombinant INF-γ (R & D Systems) at 44 U/ml). Cells were plated in plates coated with 2 μg/ml fibronectin (BD Bioscience) and incubated in tissue culture incubator at 37 °C with 5% CO_2_. Cells were progressively passed to larger plates and maintained in 60-mm tissue culture plates coated with RPE cells growth medium containing INF-γ in a tissue culture incubator at 37 °C with 5% CO_2_.

### 3-(4,5-Dimethylthiazol-2-yl)-2,5-diphenyltetrazoliumbromide (MTT) assay

The proliferation of ARPE-19 and primary RPE cells was determined by MTT assay. The cells treated with different doses of APS were plated in the 96-well plates and cultured under regular condition until they reached 80% confluence. Then, the culture medium was discarded and fresh medium containing MTT (5 mg/ml in PBS, 150 ul/well) was added followed by incubation with cells for an additional 4 h. Then, 150 ul DMSO was added per well and plate was shaken gently for 10 min to dissolve the formazan. Absorbance at 570 nm was determined using a microplate reader. Cell viability assay was performed in quadruplicate and repeated three times and the cell proliferation was presented as percentages of the value of normal cells.

### Cell culture and treatment

Human embryonic kidney cell line HEK293 was cultured according to the established protocols. Briefly, HEK293 cells were cultured DMEM supplemented with 10% fetal bovine serum (FBS), 100 U/ml penicillin, and 100 μg/ml of streptomycin. HEK293 cells were cultured at 37 °C in an incubator (Life Technologies) containing 5% CO_2_.

Human RPE cell line (ARPE-19) and rat primary RPE (PRPE) were cultured in RPE cell growth medium in 37 °C with 5% CO2. Cells were seeded in 6-well plates with the density of 1 × 10^5^ cells/well and separated for different groups. ARPE-19 cells were treated with 5 mM normal D-glucose for 6 days (NG group), 30 mM mannitol (osmotic control, OSM group), 30 mM high glucose for 6 days (HG group), or 3 days of HG followed by another 3 days of NG (HG + NG, model of metabolic memory) in the presence or absence of different dosages of APS (HG + NG + APS). For PRPE, the HG + NG and HG + NG + APS groups were changed to 2 days treated with HG followed by 4 days treated with NG in the presence or absence of APS.

### Transfection of miR-195 mimics of ARPE-19

ARPE cells treated with HG followed by NG + APS were transfected with 20 nM miR-195 negative control (NC, Genepharma) or miR-195 mimics (Genepharma) using Lipofectamine 2000 (Invitrogen) according to the manual.

### Dual luciferase reporter assay

The dual luciferase reporter vectors including wild type or mutant 3’UTR of Bcl-2 were constructed. MiR-195 NC or miR-195 mimics and reporter plasmids were co-transfected into HEK293 for 24 h. Luciferase activity was measured using the Dual Luciferase Reporter Assay System (Promega) according to the manual. The final data were the ratio of firefly fluorescent value to Renilla fluorescence value.

### Measurement of oxidative stress-related malondialdehyde (MDA) and superoxide dismutase (SOD)

The activities of MDA and SOD in RPE cells treated with HG and APS were measured using commercialized assay kits according to the manufacturer’s instructions (Nanjing Jiancheng Bioengineering Institute). Briefly, the cells were homogenized in ice-cold buffer (0.25 M sucrose, 10 mM Tris-HCl and 25 mM phenylmethylsulphonyl fluoride; pH 7.4). A portion of the homogenate was immediately measured for MDA levels. Another portion was centrifuged at 15000 g for 30 min at 4 °C, and the supernatant was decanted and assayed for SOD activity using spectrophotometer with 550 nm wavelength.

### Measurement of mitochondrial membrane potential (ΔΨm)

The ΔΨm was assessed in RPE cells using the fluorescent, lipophilic, and cationic probe 5,5′,6,6’tetrachloro-1,1′,3,3′-tetraethylbenzimidazolylcarbocyanine iodide (JC-1) according to the manufacturer’s directions. For quantitative fluorescence measurements, the cells were rinsed three times with PBS after JC-1 staining and scanned with a fluorescent microscope at an excitation wavelength of 488 nm and emission wavelength of 535 nm (mitochondrial aggregate JC-1) and excitation wavelength of 559 nm and emission wavelength of 590 nm (mitochondrial monomeric JC-1). The ΔΨm of the RPE cells in each treatment group was determined from the ratio of green fluorescence (aggregate JC-1) to red fluorescence (monomeric JC-1).

### Annexin V/PI apoptosis assay

After treatment in different groups, ARPE and PRPE cells (2 × 10^5^) were harvested and washed twice with pre-cooled PBS. The Annexin V/Dead Cell Apoptosis kit (Life Technologies) was utilized for detecting apoptotic cells. Briefly, 5 μl aliquots of Annexin V and 1 μl aliquots of Propidiumeiodide (BD Pharmingen) buffer were added into 400 μl of binding buffer. The cells were then exposed to the mixed solution for 15 min in dark at room temperature. Samples were analyzed with FACS. Then percentage of Annexin V positive cells was recorded as a measurement of cell apoptosis.

### TUNEL assay

Apoptotic cells of ARPE and PRPE were visualized by the terminal deoxynucleotidyl transferase-mediated dUTP end-labeling (TUNEL) technique using the DeadEnd Colorimetric TUNEL system (Promega). Briefly, the cells were fixed with 4% paraformaldehyde (PFA) followed by permeabilization with 0.1% Triton X-100 (Sigma-Aldrich). The apoptotic cells were stained with the TUNEL system and visualized under a fluorescence microscope according to the manufacturer’s instructions.

### Extraction of RNA and Q-PCR

Total RNA was extracted from ARPE-19 and PRPE using Trizol reagent (Invitrogen) according to the manufacturer’s protocol. 1 μg total RNA was used for reverse transcription to cDNA using reverse transcription kit (Takara). For the detection of miR-195, reverse transcription reactions were performed using the miRcute miRNA First-Strand cDNA Synthesis Kit and miRNA specific RT primer (Tiangen). Real-time qPCR assay was performed with miRcute miRNA qPCR Detection kit (SYBR Green) (Tiangen). For the detection of Bcl-2, reverse transcription reactions were performed using the FastQuant RT Kit (with gDNase) (Tiangen). Real-time qPCR assay was performed with SuperReal PreMix Plus (SYBR Green) (Tiangen). ABI Prism 7500 sequence detection system (Applied Biosystems) was used to amplify the target sequence. U6 snRNA was used as internal control for miR-195, while GAPDH was used as internal control for Bcl-2. The primers are: Rat: Bcl-2 forward: GGATCCAGGATAACGGAGGC, Bcl-2 reverse: ATGCACCCAGAGTGATGCAG; GAPDH forward: AGTGCCAGCCTCGTCTCATA, GAPDH reverse: TGAGGTCAATGAAGGGGTCG; U6 snRNA forward: CTTTGTAGGCTTCAGCGGAG, U6 snRNA reverse: ATGACGTCTGCCTTGGAGAAC. Human: Bcl-2 forward: GGGGAGGATTGTGGCCTTC, Bcl-2 reverse: CAGGGCGATGTTGTCCAC; GAPDH forward: AGACAGCCGCATCTTCTTGT, GAPDH reverse: CTTGCCGTGGGTAGAGTCAT; U6 snRNA forward: CTCGCTTCGGCAGCACA, U6 snRNA reverse: AACGCTTCACGAATTTGCGT. MiR-195 forward: GGGGAGCCAAAAGGGTCATCATCT, miR-195 reverse: GAGGGGCCATCCACAGTCTTCT.

### Western blotting assay

For the preparation of cytosolic and mitochondrial fractions, RPE cells were washed with PBS and lysed with RIPA buffer (Beyotime). The cellular extract was then centrifuged 700 g for 5 min. The supernatant was extracted and centrifuged again at 21000 g for 15 min. The supernatant was extracted as the cytosolic protein fraction. The remaining cellular pellet was washed with the same buffer and centrifuged at 21000 g for 15 min. The supernatant was discarded and the cellular pellet was washed with radioimmunoprecipitation assay buffer containing 1 mM phenylmethylsulfonyl fluoride. The washed pellet solution was centrifuged at 21000 g for 15 min, and the supernatant was extracted as the mitochondrial protein fraction. For the total cell lysates, the RPE cells were lysed with RIPA lysis buffer. The lysates were centrifuged at 13000 g for 15 min at 4 °C. The supernatants were collected and the protein concentrations were determined with a BCA Protein Assay kit (Pierce).

Equal amount (50 μg) of the protein samples were applied to 10% SDS-PAGE gels, transferred to PVDF membranes, and blocked with 5% skim milk TBST buffer. The membranes were incubated with primary antibodies including anti-Cyt-c (1:1000, Sigma-Aldrich), anti-Bcl-2 (1:1000, Cell Signaling Technology), anti-Bax (1: 1000, Cell Signaling Technology), anti-Uncleaved caspase-9 (1:2000, Cell Signaling Technology), anti-Cleaved caspase-9 (1:1000, Cell Signaling Technology), anti-Uncleaved caspase-3 (1:1000, Sigma-Aldrich), anti-Cleaved caspase-3 (1:1000, Sigma-Aldrich), anti-Uncleaved PARP (1:1000, Cell Signaling Technology), anti-Cleaved PARP (1:1000, Cell Signaling Technology), anti-COX IV (1:1000, Abcam) and anti-GAPDH (1:1000, Sigma-Aldrich) at 4 °C overnight. Then the membranes were incubate with HRP-anti-rabbit or HRP-anti-mouse secondary antibodies at room temperature for 1 h. Subsequently, bots were visualized with the enhanced chemilunimescence (Pierce) detection system.

### Statistical analysis

All experiments were performed at least for three times in triplicate, with one representative experiment shown. All data were presented as mean ± standard deviation. Statistical evaluation was performed using Student’s t test (two tailed) between two groups or one-way analysis of variance (ANOVA) followed by Tukey post hoc test for multiple comparison. The significance of all data was calculated with GraphPad Prism 5.0 software. *P* values of 0.05 or less were regarded as significant.

## Results

### The effects of APS on the survival of retinal pigment epithelium cells

**Fig. 1:**
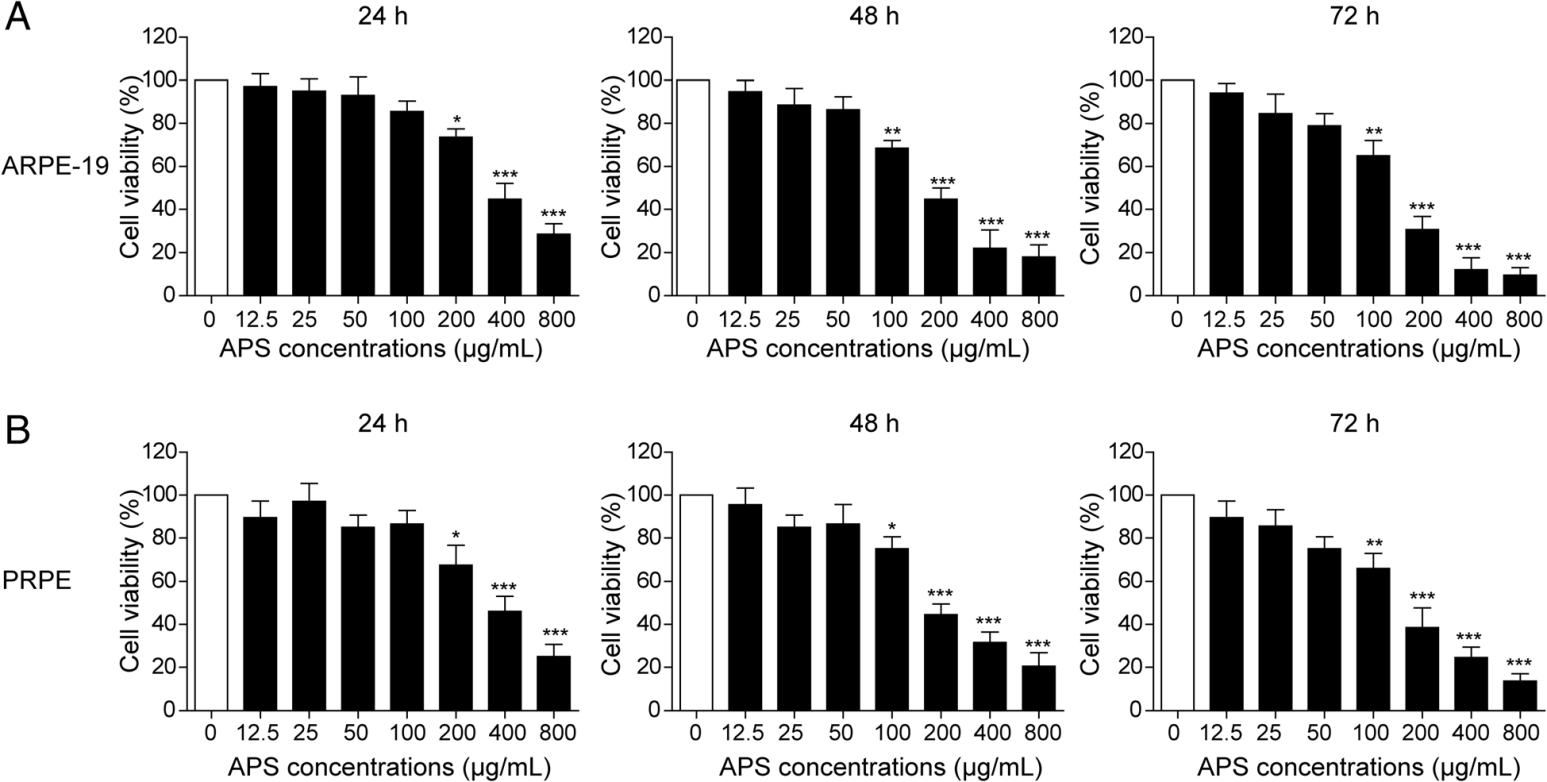
The effects of APS on the survival of retinal pigment epithelium cells. **a** Different dosages of APS were applied to treat the cultured RPE cell line (ARPE-19) for different time course. **b** Different dosages of APS were applied to treat the cultured primary RPE cells (PRPE) for different time course. Error bars represented mean ± SD. *** *p* < 0.001, ** *p* < 0.01, * *p* < 0.05

To study the effects of APS on the metabolic memory model of diabetic RPE cells, we first need to identify the suitable dosages of APS for the treatment. We examined the viability of RPE cell line (ARPE-19) and rat primary isolated RPE (PRPE) after treating with different doses of APS (12.5 μg/ml, 25 μg/ml, 50 μg/ml, 100 μg/ml, 200 μg/ml, 400 μg/ml and 800 μg/ml) for different time (24 h, 48 h and 72 h). We found the cell viability of ARPE-19 and PRPE was not significantly changed after treating with low doses of APS (0–50 μg/ml) at all the time points, whereas the high doses of APS (200–800 μg/ml) significantly decreased the cell viability (Fig. 1a and b). So the cell viability of RPE cells to APS was dose- and time-dependent. To furthest explore the function of APS on the diabetic RPE cells, we chose 3 different suitable doses (12.5 μg/ml, 20 μg/ml and 50 μg/ml) for the following experiments.

### APS inhibited the high glucose-induced upregulation of miR-195

**Fig. 2:**
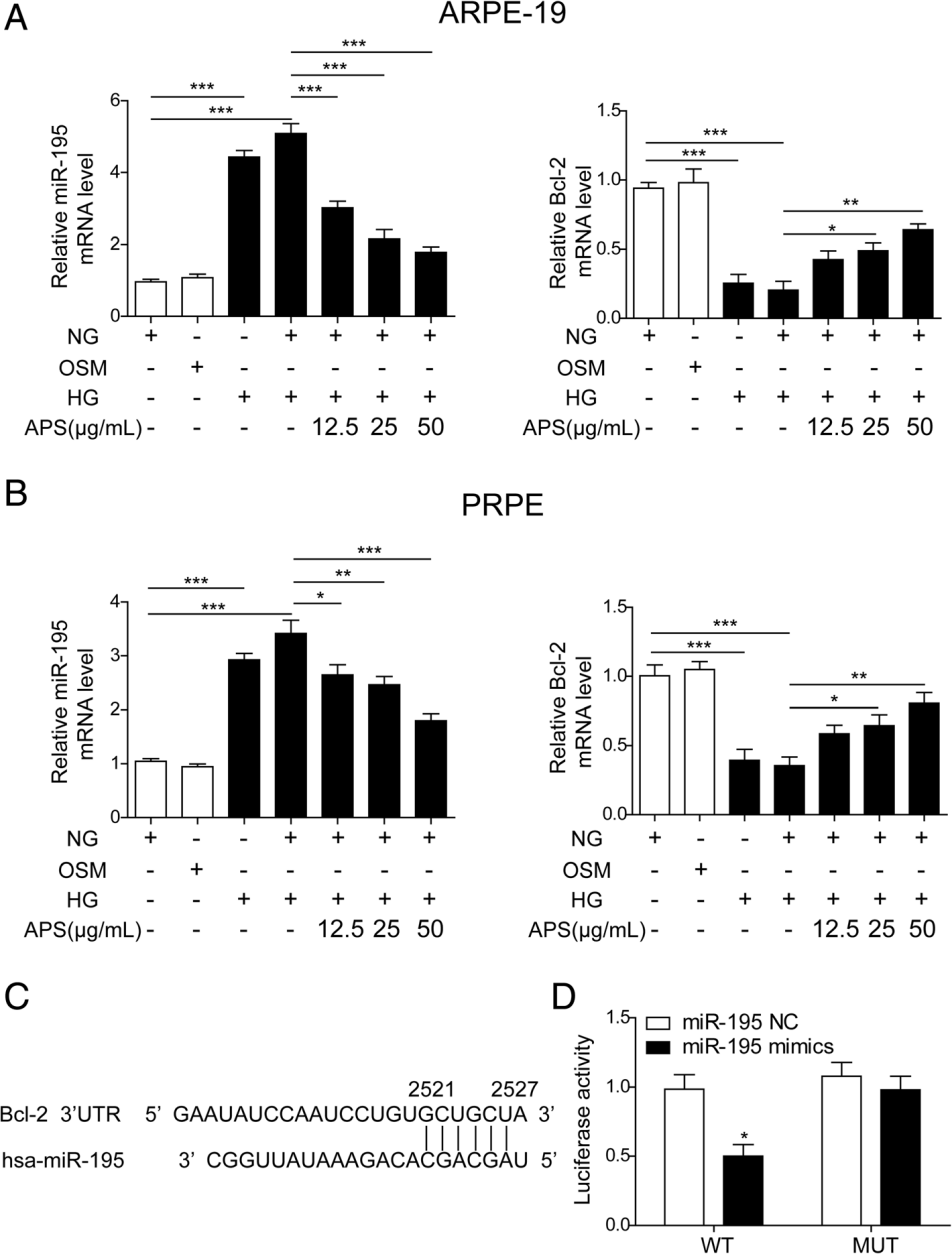
APS inhibited the high glucose-induced upregulation of miR-195. **a** Treatment with APS significantly decreased the expression of miR-195 and increased the expression of Bcl-2 which were induced by HG or HG + NG treatment in ARPE-19 cell line. **b** Treatment with APS significantly decreased the expression of miR-195 and increased the expression of Bcl-2 which were induced by HG or HG + NG treatment in PRPE cells. **c** Sequence analysis result showed miR-195 directly targeted to the 3’UTR of Bcl-2. **d** Dual luciferase reporter assay showed miR-195 repressed the luciferase activity which contained the WT Bcl-2 3’UTR, but not the mutant Bcl-2 3’UTR. Error bars represented mean ± SD. *** *p* < 0.001, ** *p* < 0.01, * *p* < 0.05

Next, we wanted to examine the effects of APS on the level of miR-195 and its target gene, Bcl-2, in the metabolic memory model of RPE cells. We treated the ARPE-19 or PRPE cells with 30 mM high glucose (HG) followed with 5 mM normal glucose (NG) in presence or absence of APS. We found HG significantly increased the expression of miR-195 and decreased the expression of Bcl-2 in ARPE-19 compared to the NG group and osmotic control (30 mM mannitol, OSM) group (Fig. 2a). The further treatment of NG followed HG maintained the upregulation of miR-195 and downregulation of Bcl-2 compared with the HG treatment which indicated the metabolic memory was happened in the HG-treated ARPE-19 cells (Fig. 2a). Then we applied different dosages of APS (25 μg/ml, 50 μg/ml and 100 μg/ml) together with NG to the HG-treated ARPE-19 cells to examine if APS can reverse the metabolic memory. We found APS significantly decreased the expression of miR-195 and increased the expression of Bcl-2 with a dosage-dependent manner (Fig. 2a). These data indicated the APS reversed metabolic memory in the HG-treated ARPE-19 cells. Consistently, we found the reverse effect was also happened in PRPE cells (Fig. 2b). To prove Bcl-2 is the directly target of miR-195, we analyzed and found the 3’UTR of Bcl-2 had a binding site for miR-195 (Fig. 2c). With dual luciferase assay, we demonstrated overexpression of miR-195 with miR-195 mimics significantly decreased the luciferase activity which containing the WT 3 ‘UTR of Bcl-2. However, if the binding site of miR-195 was mutant, the luciferase activity was abolished (Fig. 2d). These data indicated that APS might alleviate the metabolic memory of HG-treated RPE cells via regulating the expression of miR-195 and its direct target Bcl-2.

### APS rescued the oxidative stress and mitochondrial damage in RPE cells

**Fig. 3:**
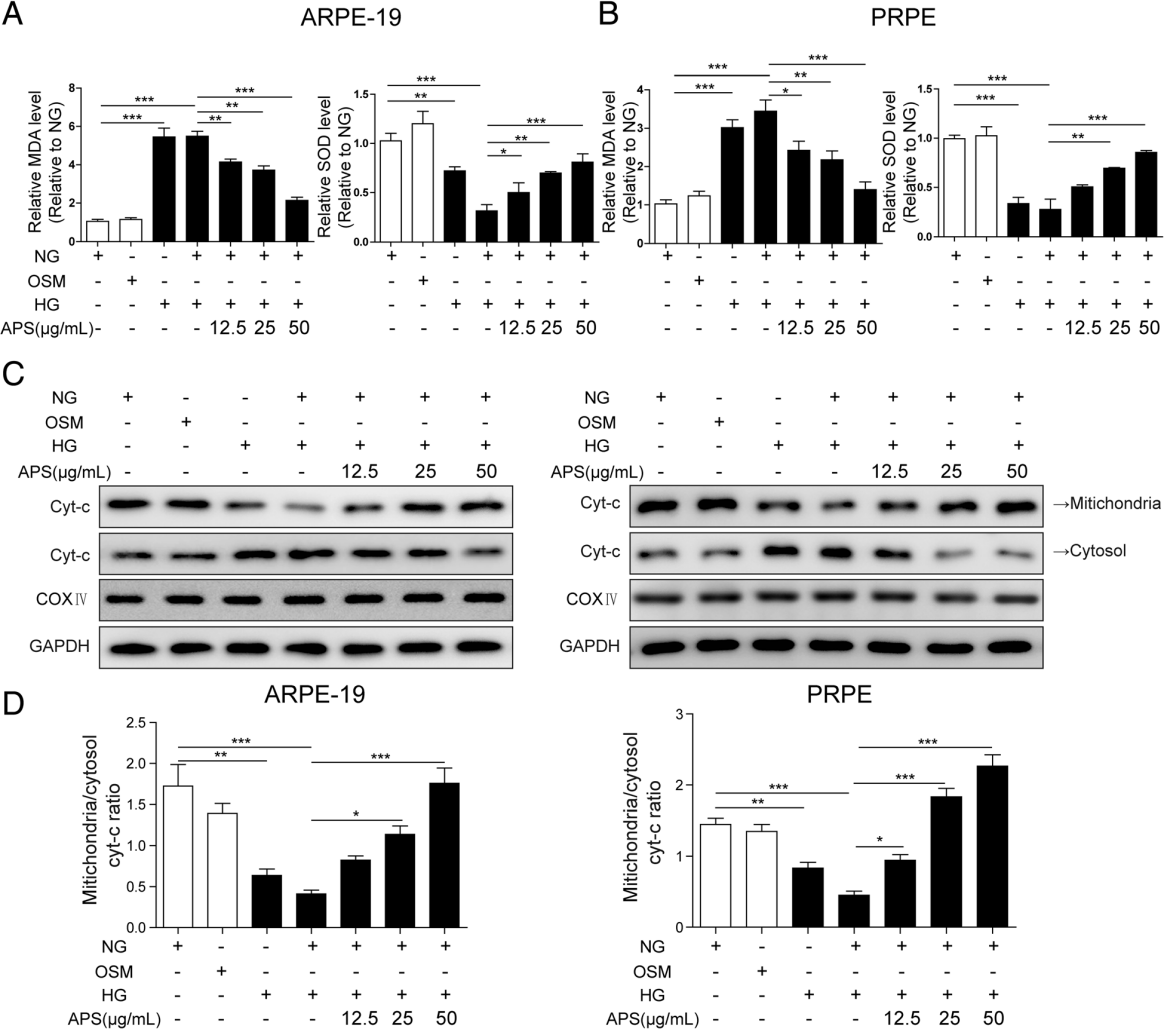
APS reversed the oxidative stress and mitochondrial damage in RPE cells induced by HG. **a** Treatment with APS significantly decreased the MDA and increased SOD level which were induced by HG or HG + NG treatment in ARPE-19 cells. **b** Treatment with APS significantly decreased the MDA and increased SOD level which were induced by HG or HG + NG treatment in PRPE cells. **c** Western blotting showed treatment with APS significantly increased the ratio of mitochondrial to cytosol cyt-c which induced by HG and HG + NG treatment in ARPE-19 and PRPE cells. **d** The statistical results of western blotting. Error bars represented mean ± SD. *** *p* < 0.001, ** *p* < 0.01, * *p* < 0.05

In the in vitro HG-induced metabolic model, the oxidative stress and mitochondrial damage are the common features of RPE cells. And Bcl-2 plays important roles in mitochondrial damage. So we wanted to know if APS could alleviate the oxidative stress and mitochondrial damage in HG-treated RPE cells. Firstly, we examined the levels of malondialdehyde (MDA) and superoxide dismutase (SOD), which were used as indexes of lipid superoxide and oxygen free radical level, after HG treatment with or without APS. We found HG significantly increased MDA and decreased SOD levels compared to the NG and OSM groups, while NG treatment followed HG treatment maintained the high MDA and low SOD levels in both APRE-19 and PRPE cells (Fig. 3a and b). This is consistent with the metabolic memory as the expressions of miR-195 and Bcl-2 (Fig. 2a and b). However, treatment with different dosages of APS together with NG after treating with HG significantly decreased the level of MDA and increased the level of SOD in a dosage-dependent manner (Fig. 3a and b). Secondly, we examined the level of mitochondrial and cytoplasmic cytochrome c (Cyt-c). The damage of mitochondria causes the release of Cyt-c from mitochondria to cytoplasm. We found HG and HG + NG treatment significantly increased the level of cytoplasmic Cyt-c while the level of mitochondrial Cyt-c was decreased which was consistent to the feature of metabolic memory (Fig. 3c and d). However, APS reversed the Cyt-c distribution and induced the ratio of mitochondrial Cyt-c to cytoplasmic Cyt-c in a dosage dependent manner which demonstrated APS protected the mitochondria damage from HG treatment (Fig. 3c and d).

**Fig. 4:**
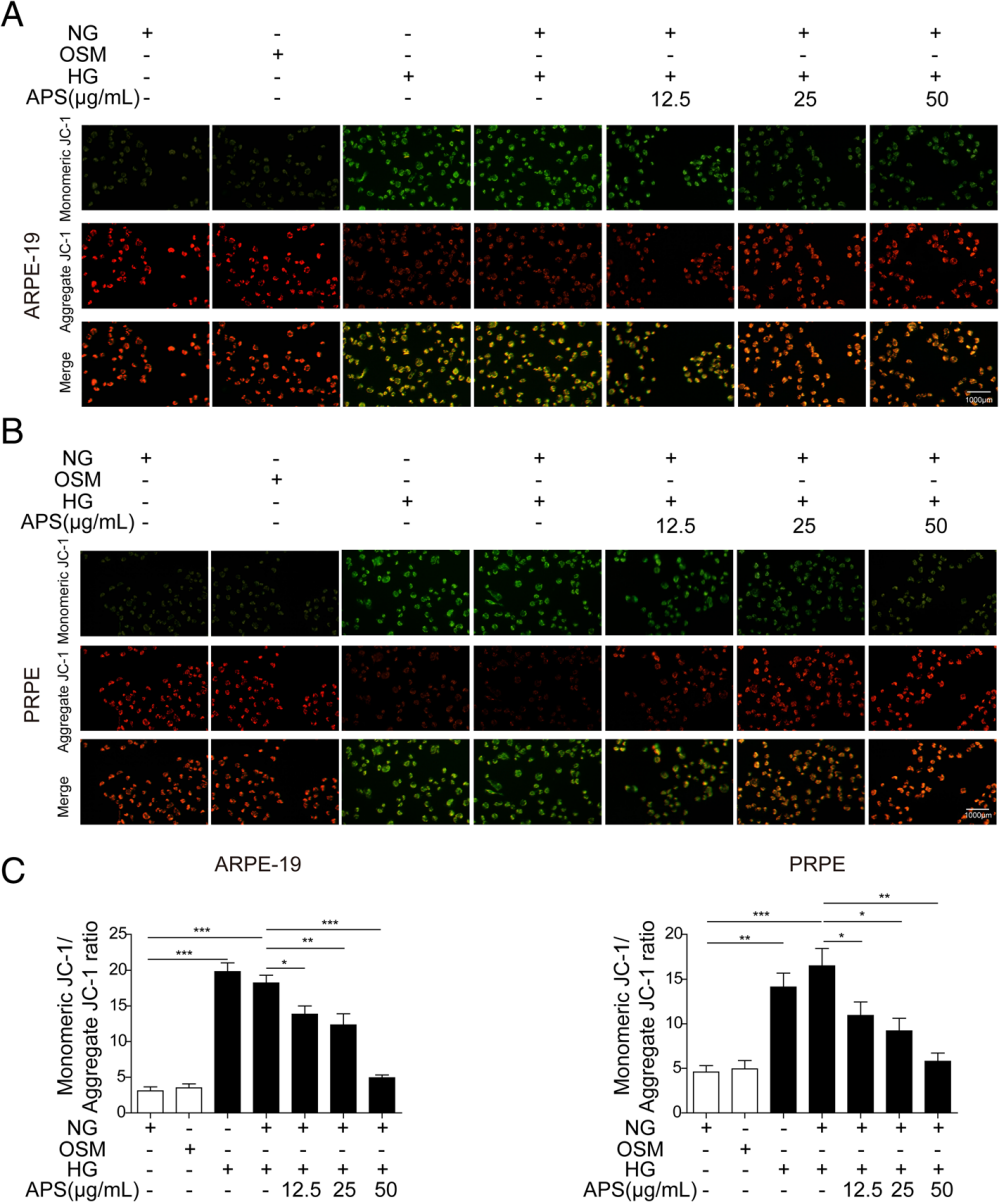
APS ameliorated the mitochondrial membrane potential induced by HG in RPE cells. **a** Immunostaining showed treatment with APS significantly decreased the ratio of monomeric to aggregate JC-1 which was induced by HG or HG + NG treatment in ARPE-19 cells. **b.** Immunostaining showed treatment with APS significantly decreased the ratio of monomeric to aggregate JC-1 which was induced by HG or HG + NG treatment in PRPE. **c** The statistical results of the staining in A and B. Error bars represented mean ± SD. *** *p* < 0.001, ** *p* < 0.01, * *p* < 0.05

Then we examined the mitochondrial damage after HG treatment with or without APS via detecting the monomeric and aggregate JC-1, which was used to detect the mitochondrial membrane potential (MMP) (See Materials and Methods). The increased ratio of monomeric JC-1 (green) to aggregate JC-1 (red) indicates the depolarization of the mitochondria, which demonstrates the mitochondrial damage (Huang et al. 2013). We found HG and HG + NG treatment significantly increased the monomeric JC-1 level and the ratio of monomeric JC-1 to aggregate JC-1 compared to NG and OSM treated ARPE-19 and PRPE cells, which indicated the mitochondrial damage of RPE cells under HG with metabolic memory (Fig. 4a to c). Whereas treatment with different dosages of APS with NG after HG treatment significantly decreased the expression of monomeric JC-1 and this ratio which demonstrated the APS alleviated mitochondrial damage (Fig. 4a to c). Meanwhile, we found higher dosage of APS decreased monomeric JC-1 more, which indicated the effect of APS on the mitochondrial damage is dose-dependent (Fig. 4c). All of these data indicated HG led to the oxidative stress and mitochondrial damage with metabolic memory which could be rescued by APS in a dosage-dependent manner.

### APS rescued the apoptosis induced by HG in RPE cells

**Fig. 5:**
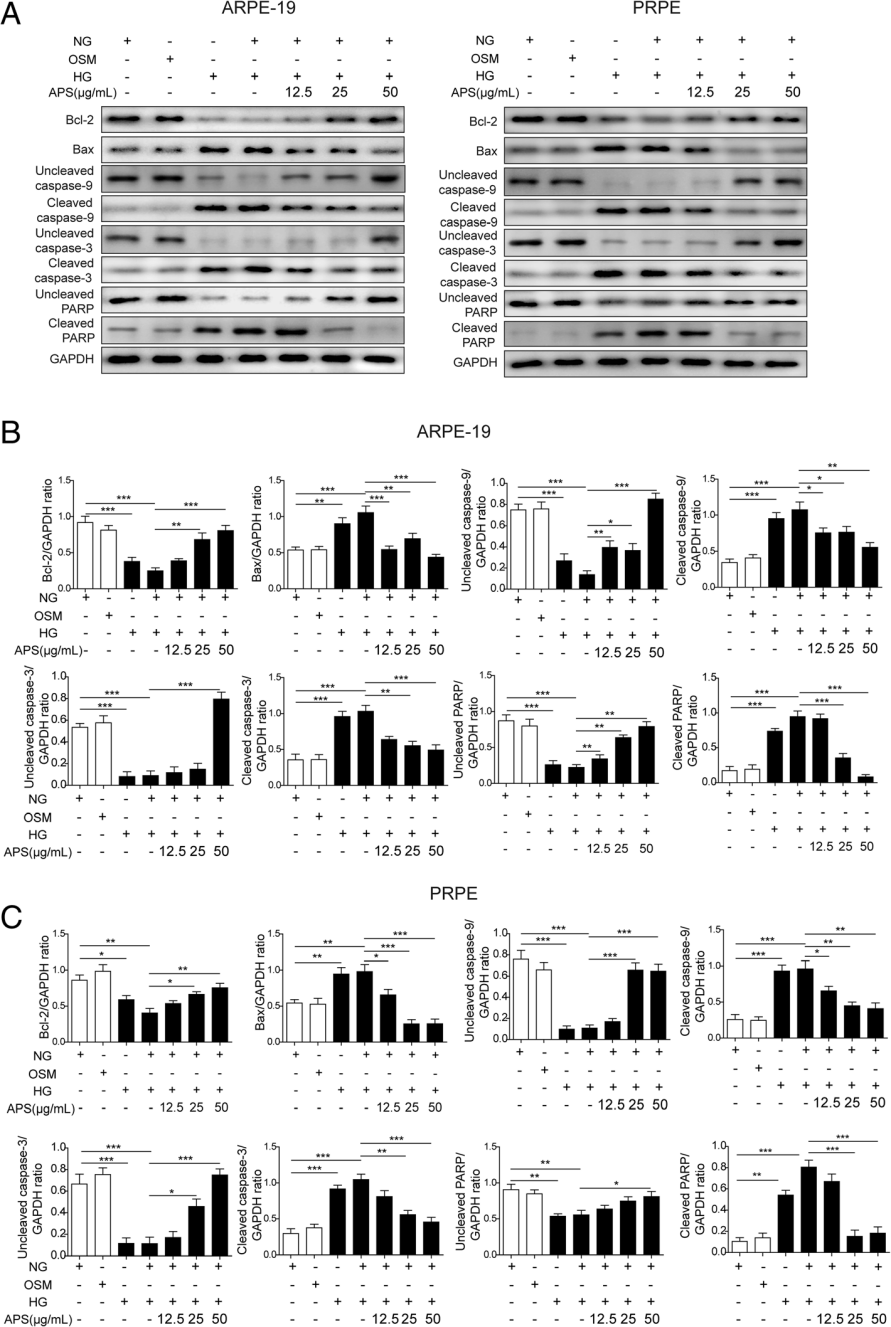
APS decreased the RPE cells apoptosis via mitochondrial apoptotic pathway. **a** Western blotting showed treatment with APS significantly decreased the expression of Bax, cleaved caspase-9, cleaved caspase-3 and cleaved PARP which were induced by HG and HG + NG treatment, while increased the expression of Bcl-2 and uncleaved form of caspase-9, caspase-3 and PARP which were repressed by HG and HG + NG treatment in APRE-19 and PRPE cells. **b** and **c.** The statistical results of western blotting in APRE-19 and PRPE cells. Error bars represented mean ± SD. *** *p* < 0.001, ** *p* < 0.01, * *p* < 0.05

**Fig. 6:**
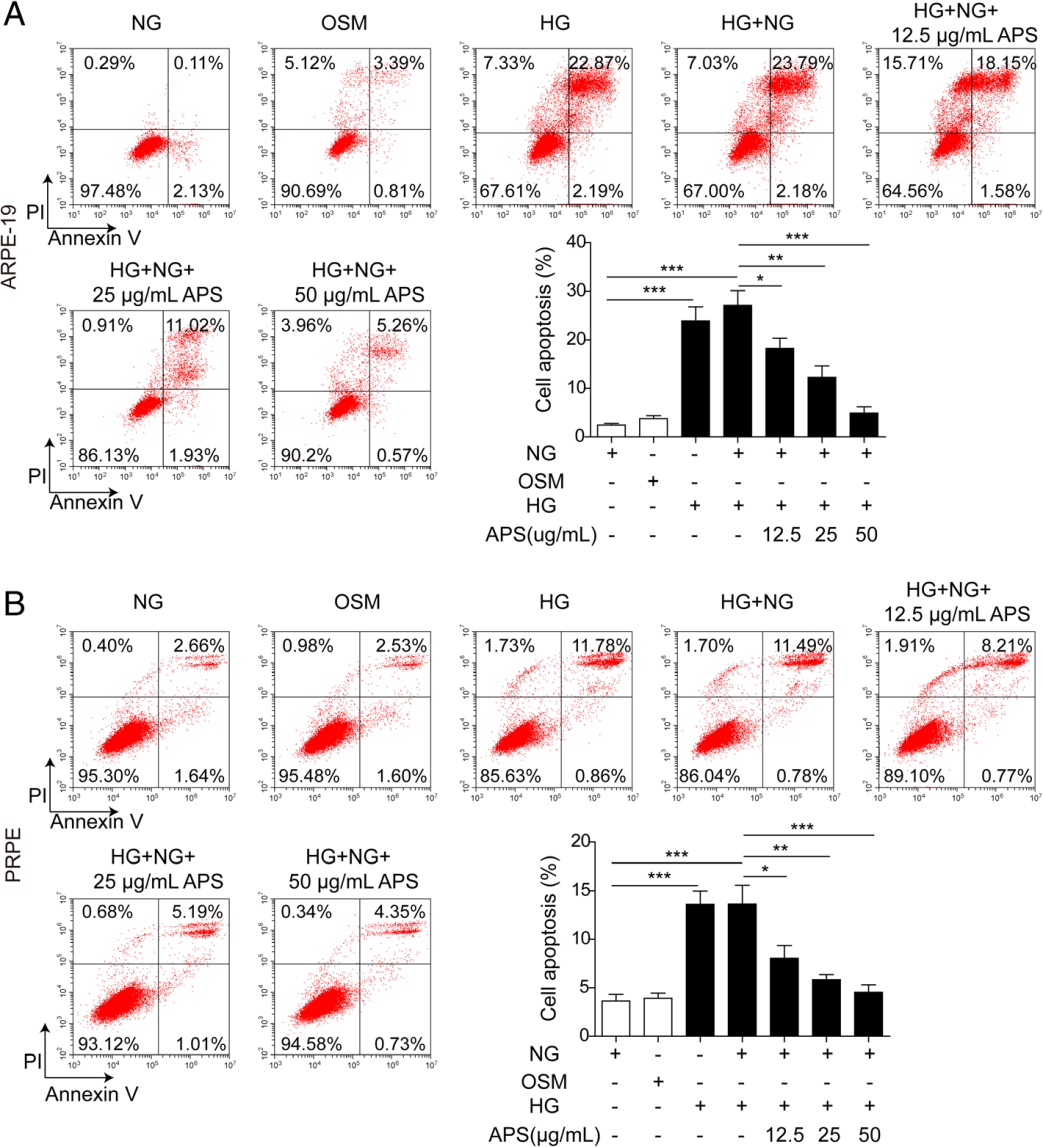
APS rescued the apoptosis in metabolic memory model by flow cytometry. **a** Flow cytometry showed treatment with APS significantly decreased the cell apoptosis induced by HG and HG + NG treatment in APRE-19 cells. **b** Flow cytometry showed treatment with APS significantly decreased the cell apoptosis induced by HG and HG + NG treatment in PRPE cells. Error bars represented mean ± SD. *** *p* < 0.001, ** *p* < 0.01, * *p* < 0.05

**Fig. 7:**
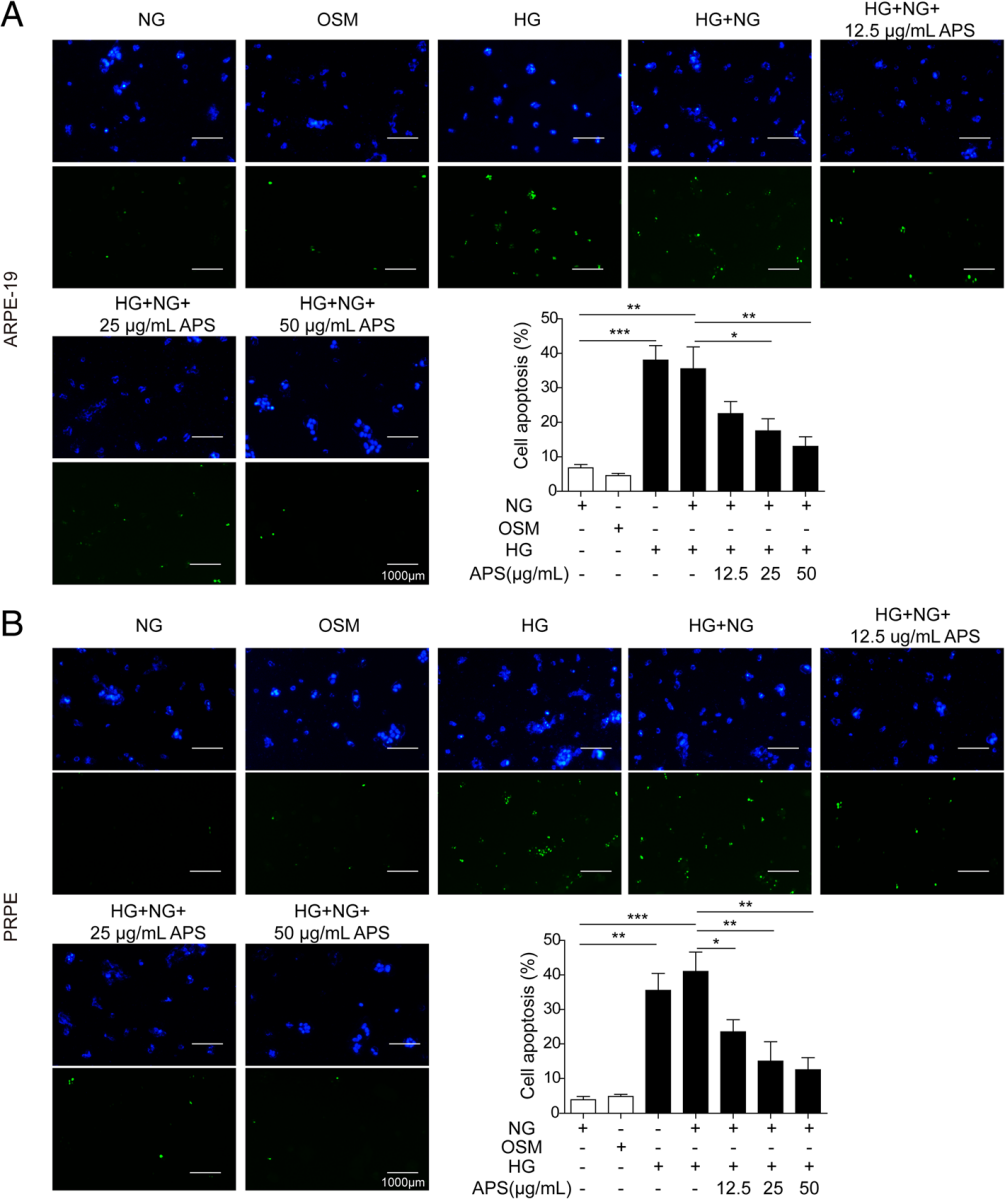
APS rescued the apoptosis in metabolic memory model by TUNEL staining. **a** TUNEL staining showed treatment with APS significantly decreased the TUNEL positive cells induced by HG and HG + NG treatment in APRE-19 cells. **b** TUNEL staining showed treatment with APS significantly decreased the TUNEL positive cells induced by HG and HG + NG treatment in PRPE cells. Error bars represented mean ± SD. *** *p* < 0.001, ** *p* < 0.01, * *p* < 0.05

The dysfunction of mitochondria may cause the cell apoptosis via Bcl-2/Bax/Caspase-9 signaling pathway (Alarifi et al. 2017; Li et al. 2017). So we next examined if APS can rescue the HG-induced RPE cells apoptosis. Via western blotting, we found HG and HG + NG treatment significantly decreased the expressions of Bcl-2 and uncleaved PARP and increased the expressions of Bax, cleaved caspase-9 and cleaved caspase-3 compared to NG and OSM treated groups in both ARPE-19 and PRPE cells (Fig. 5a to c). Meanwhile, the treatment of HG and HG + NG significantly increased the cleaved PARP and decreased the uncleaved caspase-9 and caspase-3 (Fig. 5a to c). But the treatment of different dosages of APS with NG followed the HG treatment significantly reversed the expressions of these proteins which indicated APS inhibited the apoptotic pathway induced by HG treatment in a dosage-dependent manner (Fig. 5a to c). Then we applied flow cytometry to further prove APS indeed decreased the cell apoptosis. We found the apoptosis was increased by HG (about 25.06% in APRE-19, 12.64% in PRPE) and HG to NG (about 25.97% in APRE-19, 12.27% in PRPE) treatment compared to NG (about 2.24% in APRE-19, 4.30% in PRPE) and OSM (about 4.20% in APRE-19, 4.13% in PRPE) groups, which was inhibited by different dosages of APS (about 19.73% for 12.5 μg/ml APS in ARPE-19, 12.95% for 25 μg/ml APS in ARPE-19, 5.83% for 50 μg/ml APS in ARPE-19, 8.98% for 12.5 μg/ml APS in PRPE, 6.20% for 25 μg/ml APS in PRPE, 5.08% for 50 μg/ml APS in PRPE) (Fig. 6a and b). Furthermore, we examined the apoptotic cells via TUNEL stainning. Indeed, the APS treatment significantly rescued the apoptosis induced by HG treatment (Fig. 7a and b). These results demonstrated APS rescued the RPE cells apoptosis caused by HG-induced metabolic memory of mitochondrial damage.

### MiR-195 increased the oxidative stress and mitochondrial damage inhibited by APS in ARPE-19 cells

**Fig. 8:**
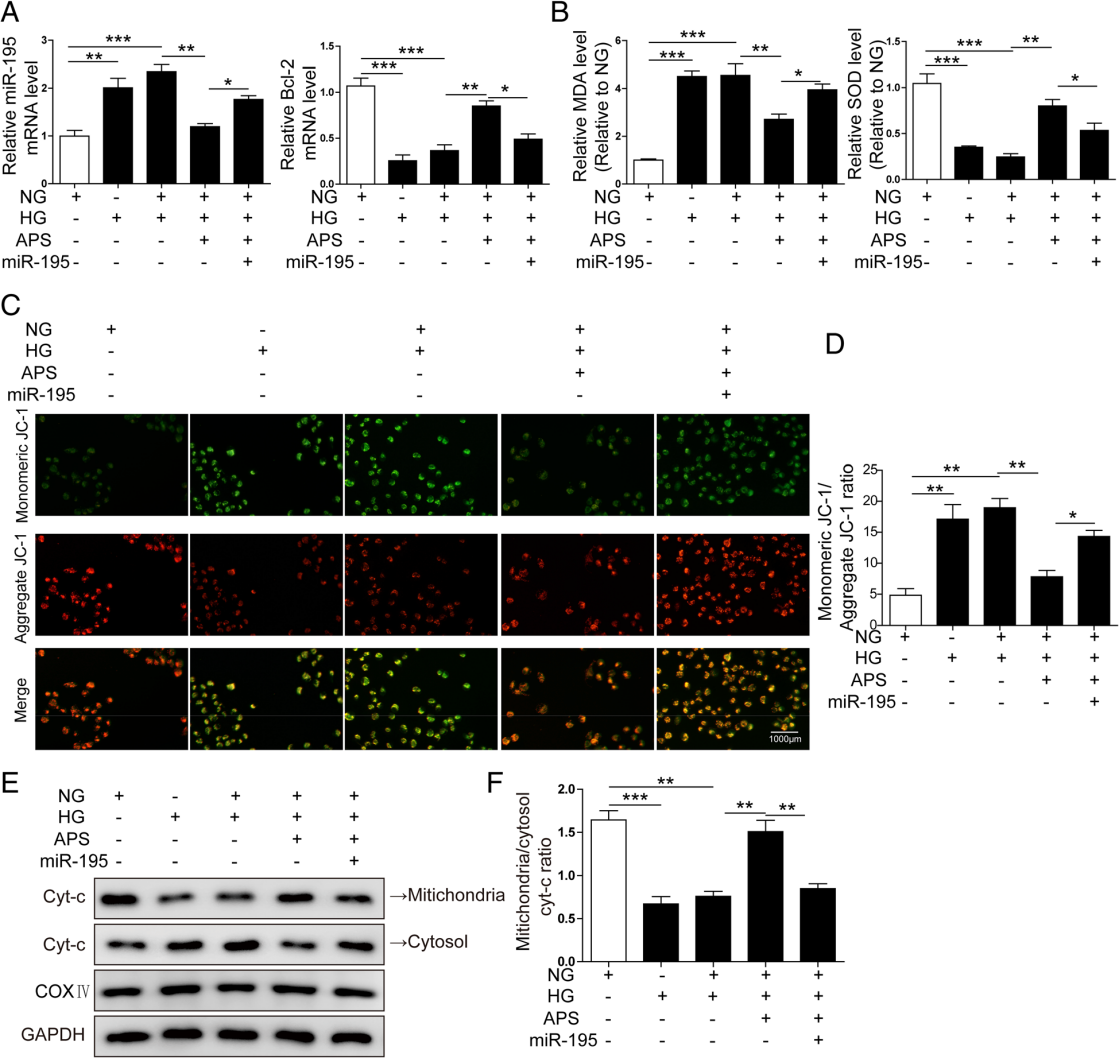
Overexpression of miR-195 increased the oxidative stress and mitochondrial damage in ARPE-19 cells. **a** MiR-195 mimics significantly increased the miR-195 level and decreased the Bcl-2 level which were modulated by APS treatment in HG and HG + NG treated ARPE-19 cells. **b** MiR-195 mimics increased the MDA and decreased SOD levels which were regulated by APS in HG and HG + NG treated ARPE-19 cells. **c** Immunostaining showed miR-195 mimics significantly increased the ratio of monomeric (Green) to aggregate (Red) JC-1 which was decreased by APS in HG or HG + NG treated ARPE-19 cells. **d** The statistical results of mitochondrial membrane potential in C. **e** Western blotting showed miR-195 mimics significantly decreased the ratio of mitochondrial to cytosol cyt-c which was increased by APS in HG and HG + NG treated ARPE-19 cells. **f** The statistical results of ratio of mitochondrial to cytosol cyt-c. Error bars represented mean ± SD. *** *p* < 0.001, ** *p* < 0.01, * *p* < 0.05

We found HG induced the expression of miR-195 and decreased the expression of Bcl-2 in RPE cells, which could be repressed by APS (Fig. 2a and b). Next we verified the benefit effect of APS was or not through repressing the expression of miR-195 in the HG-induced ARPE-19 cells. We overexpressed miR-195 together with APS treatment after HG treatment. The level of miR-195 was increased by miR-195 mimics compared with the APS treated group (Fig. 8a). Meanwhile, the direct target Bcl-2, which was upregulated by APS treatment, was repressed again after transfected with miR-195 mimics, which indicated miR-195 mimics efficiently upregulated the miR-195 level even in APS-treated ARPE-19 cells (Fig. 8a). Then we examined the oxidative stress after overexpression of miR-195 via measuring the levels of MDA and SOD. The results showed miR-195 reversed the levels of MDA and SOD regulated by APS in HG-treated ARPE-19 cells (Fig. 8b). Next, we examined the function of miR-195 on mitochondrial damage in HG and APS-treated ARPE-19 cells. The results showed the ratio of monomeric JC-1 to aggregate JC-1, which was repressed by APS in HG-treated ARPE-19, was increased again by miR-195 mimics (Fig. 8c and d). Second, we found that the cytoplasmic Cyt-c was increased while the mitochondrial Cyt-c was decreased when miR-195 was overexpressed in HG and APS-treated ARPE-19 (Fig. 8e and f). These data indicated the alleviated mitochondrial function by APS was damaged by miR-195. All these data demonstrated the alleviation of mitochondrial damage and metabolic memory by APS was indeed through repressing the expression of miR-195.

### MiR-195 reversed the inhibition of the apoptosis by APS in ARPE-19 cells

**Fig. 9:**
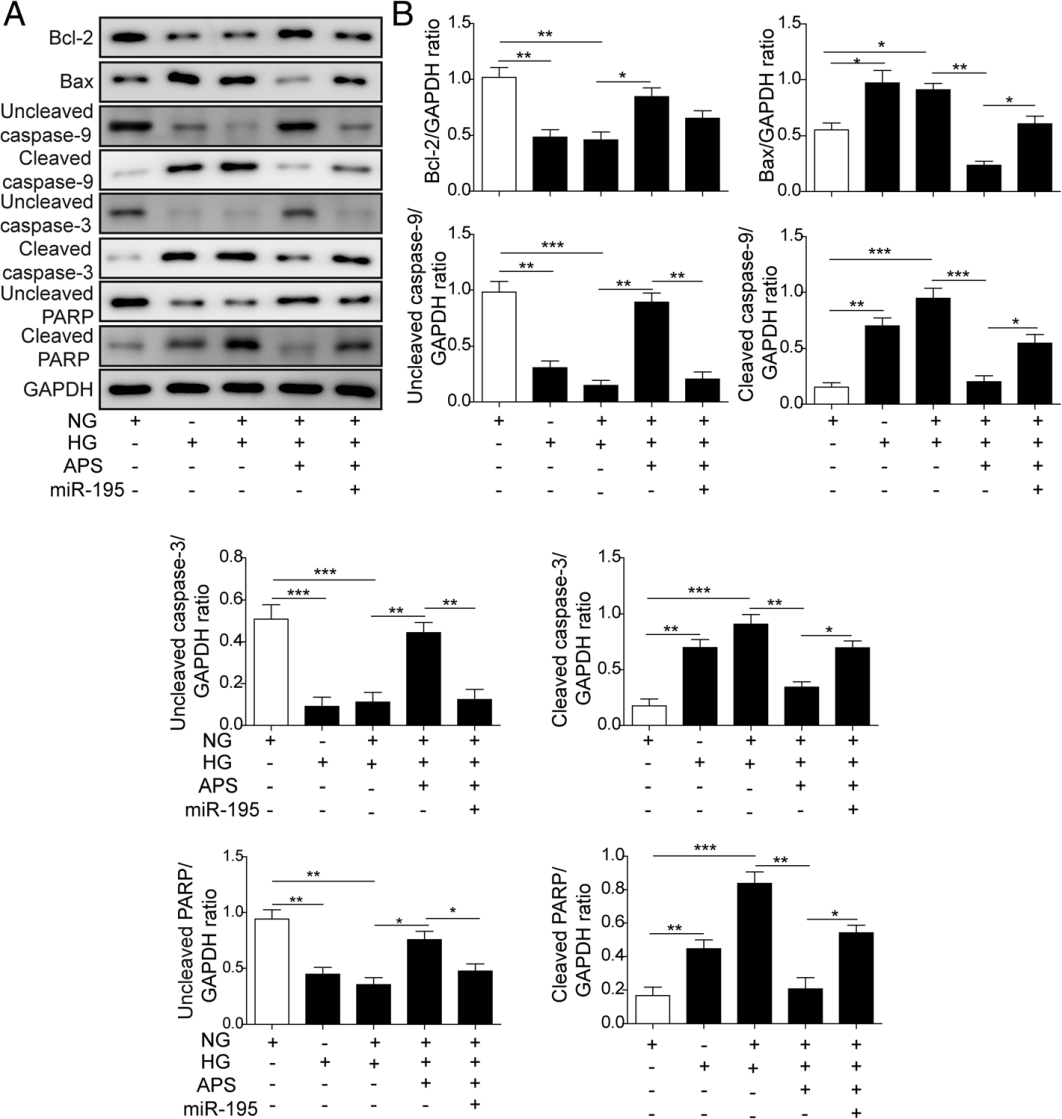
MiR-195 activated mitochondrial apoptotic pathway in ARPE-19 cells. **a** Western blotting showed miR-195 mimics significantly increased the expressions of Bax, cleaved caspase-9, cleaved caspase-3 and cleaved PARP while decreased the expressions of Bcl-2 and uncleaved form of caspase-9, caspase-3 and PARP compared to the APS treated ARPE-19 cells. **b**The statistical results of western blotting in A. Error bars represented mean ± SD. *** *p* < 0.001, ** *p* < 0.01, * *p* < 0.05

**Fig. 10:**
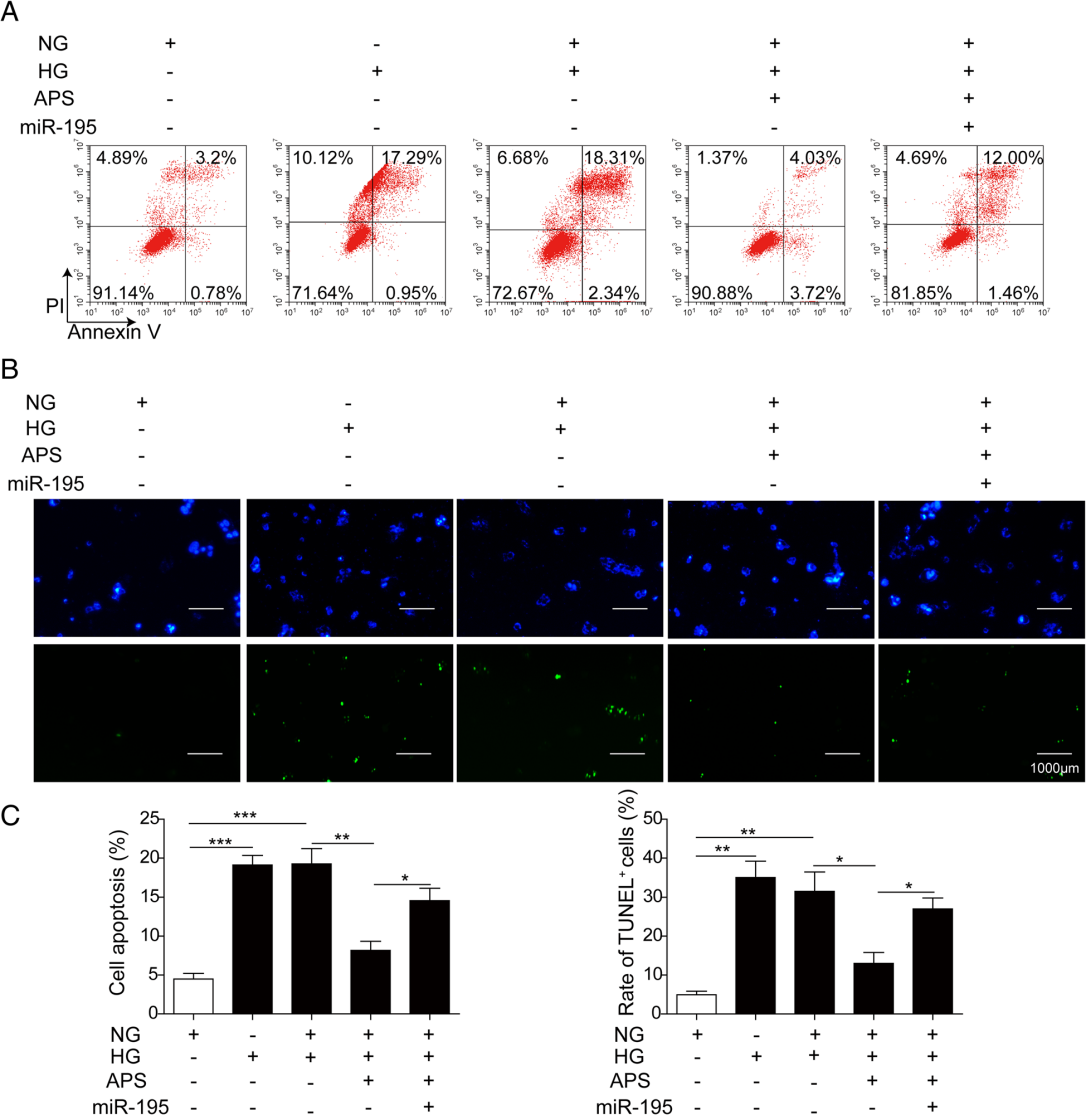
Overexpression of miR-195 enhanced the cell apoptosis in ARPE-19 cells. **a** Flow cytometry showed miR-195 increased the cell apoptosis compared to the APS treated ARPE-19 cells. **b** TUNEL staining showed miR-195 increased the TUNEL positive cells compared to the APS treated ARPE-19 cells. **c** The statistical results of flow cytometry and TUNEL staining in A and B. Error bars represented mean ± SD. *** *p* < 0.001, ** *p* < 0.01, * *p* < 0.05

Next, we examined the function of miR-195 on the cell survival of ARPE-19 cells which were treated by HG with or without APS. First, we detected the apoptotic signaling pathway via western blotting after overexpressing miR-195. The results showed the decreased expression of Bax, cleaved caspase-9 and cleaved caspase-3 by APS in HG-treated ARPE-19 were increased again by miR-195, while the expression of Bcl-2 and uncleaved PARP was decreased by miR-195 (Fig. 9a and b). Meanwhile, miR-195 increased the expression of cleaved PARP, and decreased the uncleaved caspase-9 and caspase-3 (Fig. 9a and b). Second, we did the flow cytometry to detect the cell apoptosis. The results showed overexpression of miR-195 significantly increased the cell apoptosis which was rescued by APS in the HG-treated ARPE-19 cells (Fig. 10a and c). Third, we stained the ARPE-19 with TUNEL to examine the cell apoptosis. We found the TUNEL positive cells were significantly increased after overexpressing miR-195 in the APS-rescued ARPE-19 (Fig. 10b and c). All of these data demonstrated the benefit effect of mitochondrial damage-induced apoptosis of APS in the RPE was through downregulating the level of miR-195.

## Discussion

Diabetic retinopathy (DR) is a major complication of diabetes leading to the vision impairment and blindness (Frank 2004). Metabolic memory, which indicates hyperglycemia has long-lasting detrimental consequences in diabetic patients even after the strict glycemic control, and contributes to the development of DR (Kowluru 2013; Santos and Kowluru 2011; Zhao et al. 2015). Targeting to metabolic memory may provide a new strategy for the treatment of DR. Lots of molecular pathways are involved in the metabolic memory. Increase of the inflammatory mediators and oxidative stress are the metabolic abnormalities associated with the metabolic memory (Kowluru 2003; Kowluru et al. 2004b; Kowluru et al. 2010). Increased production of oxidative stress can modulate other signaling pathways, like PKC, NF-κB, AGE and so on, to further produce ROS leading to the metabolic memory (Brownlee 2005). The increased nuclear transcriptional factor κB (NF-κB) continues to be increased, indicating the signaling cascade does not turn off even under normal glycemia (Kowluru et al. 2004b). Furthermore, mitochondrial dysfunction is also involved in metabolic memory (Tewari et al. 2012; Santos and Kowluru 2011; Madsen-Bouterse et al. 2010a; Madsen-Bouterse et al. 2010b). In DR, retinal mitochondria is dysfunctional and membrane potential is impaired. The damaged mitochondrial DNA impairs the biogenesis of mitochondria which contributes to the metabolic memory. This vicious cycle continues to self-propagate and doesn’t turn off even after the condition is changed to the normal glucose. APS is a Chinese traditional medicine which can suppress the mitochondrial damage and prevent the diabetes (Liu et al. 2013; Dun et al. 2016). In the current study, we found APS suppressed high glucose-induced metabolic memory in retinal pigment epithelial cells through inhibiting mitochondrial dysfunction-induced apoptosis by regulating miR-195. So APS may be used to treat DR via suppressing the metabolic memory. This will provide a new therapeutic way for DR.

To examine the effects of APS on the metabolic memory, we chose three dosages of APS based on the cell toxicity on RPE cells (Fig. 1). We found the higher dosages APS led to the cell death and chose three safe concentrations to perform the next experiments. Then we established a model of metabolic memory by treating the RPE cells with NG after HG treatment (Fig. 2). MiR-195 is a miRNA which is highly expressed in the diabetes (Ortega et al. 2014; Herrera et al. 2010). MiR-195 plays important roles in diabetes and DR. MiR-195 regulates the tissue damage in DR via targeting to Sirtuin 1 (Mortuza et al. 2014). In diabetic rats, miR-195 is involved in the oxidative stress-induced retinal endothelial cell injury via targeting to mitofusin 2 (Zhang et al. 2017). Furthermore, miR-195 regulates the mitochondrial damage via targeting to mitofusin 2 in hippocampal neurons (Zhang et al. 2016). In cardiomyocytes, overexpression of miR-195 may promote the cell apoptosis via Bcl-2 and inducing the mitochondrial apoptotic pathway (Gao et al. 2016). Thus we verified the metabolic memory via examining the level of miR-195. We found miR-195 was upregulated after the treatment of HG which was maintained even after withdrawing of HG. These data is consistent with previous reports about the function of miR-195 in DR and the successful model of metabolic memory. Treatment with APS significantly decreased the expression of miR-195 and increased the expression of miR-195 targeting gene, Bcl-2 (Fig. 2). These data suggested APS reversed the gene expression on this metabolic memory model. In our data, we found the effects of APS were dosage-dependent and higher dosages of APS were more efficient to alleviate the increase of miR-195 expression. All of these data suggested it was very important to map the dosage for the further usage of APS. Furthermore, in our results, we firstly found and verified Bcl-2 was the direct target of miR-195 in the regulation of metabolic memory via dual-luciferase reporter assay (Fig. 2c and d). Our results firstly demonstrated miR-195 regulated the metabolic memory via Bcl-2. However, studies have found miR-195 targeted to many other genes to regulate the diabetic retinopathy. For example, miR-195 accelerated retinal endothelial injury by targeting to mitofusin 2 (Zhang et al. 2017), miR-195 regulated Sirtuin 1-mediated tissue damage in DR (Mortuza et al. 2014). It will be interesting to investigate if other targets of miR-195 are involved in the metabolic memory.

In DR, increased oxidative stress and mitochondrial dysfunction lead to the cell apoptosis which finally causes the blindness (Kowluru 2013). Hyperglycaemia induces oxidative stress in RPE to promote the development of choroidal neovascularization which may lead to the blindness (Li et al. 2012). Oxidative stress in RPE cells can disrupt the blood-retinal barrier which causes the pathogenesis of DR (Bailey et al. 2004). Retinal mtDNA was damaged in diabetes which promoted the development of DR (Madsen-Bouterse et al. 2010a). During the development of DR, mitochondria was damaged including morphology changes, altered membrane potential and impaired oxygen consumption (Kowluru 2005; Kanwar et al. 2007; Trudeau et al. 2010). Furthermore, metabolic memory of RPE was suggested in previous study (Ferrington et al. 2017). As APS can reverse the Bcl-2 expression of RPE cells treated by HG or HG + NG, it’s important to examine if APS can reverse the mitochondrial damage-induced cell apoptosis caused by the HG treatment. We examined the MDA and SOD levels and found treatment of HG and HG + NG significantly increased the oxidative stress. The results of mitochondrial membrane potential and the distribution of Cyt-c in mitochondria and cytosol demonstrated the damage of mitochondria. The alteration expression of apoptosis related proteins, the increased apoptotic cells by FACS and TUNEL staining also demonstrated treatment of HG and HG + NG significantly increased the apoptosis of RPE cells (Figs. 3, 4, 5, 6 and 7). However, APS treatment reversed these changes to decrease the mitochondrial damage-induced apoptosis signaling pathways, increased the survival pathway and preserved the survival of RPE cells (Figs. 3, 4, 5, 6 and 7). These results demonstrated APS alleviated the metabolic memory via regulating mitochondrial damage-induced apoptosis via Bcl-2, which may be useful for the treatment of DR.

Since miR-195 was upregulated in the metabolic memory model and APS repressed the level of miR-195, we verified if the repression of miR-195 by APS in the metabolic memory RPE model was functional via re-introduction of miR-195 in ARPE-19 cells. We overexpressed the miR-195 in ARPE-19 cells and found overexpression of miR-195 increased the MDA and SOD levels again which were inhibited by APS, and the mitochondrial membrane potential was impaired again by overexpressing of miR-195. Furthermore, overexpression of miR-195 increased the expression of apoptotic proteins and the apoptotic cells which was inhibited by APS previously (Figs. 8, 9 and 10). These data demonstrated miR-195 played critical roles in the effects of APS on metabolic memory in RPE cells.

## Conclusions

In conclusion, we found APS ameliorated the metabolic memory in HG-treated RPE cells to rescue the mitochondrial damage-induced cell apoptosis via repressing miR-195. MiR-195 was proved to contribute to the function of APS by targeting Bcl-2 in this process. Our data identified APS may be an efficient drug to treat the metabolic memory of DR. This may provide a new avenue for the treatment of DR.

## Abbreviations

AMD: Age-related macular degeneration
APS: Astragalus polysaccharides
Cyt-c: Cytochrome c
DCCT: Diabetes Control and Complications Trial
DR: Diabetic retinopathy
EDIC: Epidemiology of Diabetes Interventions and Complications
HG: High glucose
MDA: Malondialdehyde
MMP: Mitochondrial membrane potential
NC: Negative control
NF-κB: Nuclear transcriptional factor κB
NG: Normal glucose
PRPE: Primary RPE cells
RPE: Retinal pigment epithelium
SOD: Superoxide dismutase
UTR: Untranslated region
